# A Novel Fixed-Dose Activated Prothrombin Complex Concentrate Regimen for Warfarin-Associated Hemorrhages: A Retrospective Cohort Comparison of Two Regimens †

**DOI:** 10.3390/pharmacy14050121

**Published:** 2026-08-19

**Authors:** Francisco Ibarra, Evan Cheng, Benjamin Falkenstein

**Affiliations:** 1 Community Regional Medical Center, Department of Pharmacy Services, 2823 Fresno St., Fresno, CA 93715, USA; 2 College of Osteopathic Medicine, California Health Sciences University, 2500 Alluvial Ave, Clovis, CA 93611, USA; 3 Department of Emergency Medicine, University of California San Francisco at Fresno, 155 N Fresno St., Fresno, CA 93701, USA

**Keywords:** warfarin reversal, hemorrhage, anticoagulant, FEIBA, PCC, prothrombin complex concentrate

## Abstract

The optimal fixed-dose strategy for managing warfarin-associated hemorrhages remains unknown and few studies have evaluated the use of Factor VIII Inhibitor Bypass Activity (FEIBA). This retrospective cohort study’s primary efficacy outcome was the percentage of patients who achieved a post-FEIBA INR ≤ 1.5 following receipt of the old and new dosing regimens. In the old group patients received 500 or 1000 units for an INR < 5 or ≥5, respectively. In the new group patients received 1000, 1500, or 2000 units for an INR < 5, 5–9.9, or ≥10, respectively. Eighteen patients were included in each group. The median (IQR) pre-FEIBA INR in the old and new groups was 6.1 (3.1–12.9) and 5.3 (3.4–13.0), respectively [difference: −0.8 (95 CI%, −7.2 to 6.5)]. The median (IQR) post-FEIBA INR in the old and new groups was 1.6 (1.4–2.1) and 1.4 (1.2–1.5), respectively [difference: −0.2 (95% CI: −0.6 to 0.0)]. A post-FEIBA INR ≤ 1.5 was achieved in 14 (78%) patients in the new group and 9 (50%) patients in the old group (relative risk: 1.56; 95% CI, 0.91 to 2.7; *p* = 0.16). The post-FEIBA INR values in the patients who did not achieve a post-FEIBA INR ≤ 1.5 in the new group were 1.6, 1.6, 1.8, and 2.4. Among patients with a baseline INR ≥ 10, significantly more patients in the new group achieved a post-FEIBA INR ≤ 1.5 compared to the old group (86% vs. 17%, respectively). These findings suggest that the new FEIBA dosing regimen may improve INR reversal compared with the previous regimen, particularly among patients with a baseline INR ≥ 10, but larger studies are needed to confirm this observation.

## 1. Introduction

Patients presenting with major warfarin-associated hemorrhages are commonly treated with a combination of vitamin K and four-factor prothrombin complex concentrate (PCC) to rapidly reverse anticoagulation and promote hemostatic control [1]. Vitamin K replenishes vitamin K stores, enabling the production of new clotting factors, but has a delayed onset of action [2,3,4]. Unlike vitamin K, PCC facilitates rapid international normalized ratio (INR) correction through the direct replacement of coagulation factors. Kcentra and Balfaxar are the only FDA approved and guideline recommended PCC for managing warfarin-associated hemorrhages; however, Factor VIII Inhibitor Bypass Activity (FEIBA) is commonly used in practice due to its demonstrated efficacy [5,6]. In comparison to Kcentra and Balfaxar, FEIBA is FDA approved for use in hemophilia A and B patients with inhibitors and contains activated factor VII.

Low fixed-dose PCC regimens are increasingly replacing manufacturer-recommended, variable-dose regimens due to their comparable efficacy and reduced costs [7,8,9,10,11,12,13,14,15,16,17,18,19,20,21,22,23]. However, outcomes among proposed fixed-dose strategies vary and the optimal fixed-dose regimen remains unknown [24]. Despite the widespread adoption of direct oral anticoagulants, multiple professional societies continue to recommend warfarin as first-line therapy in select patient populations (i.e., mechanical heart valves, triple positive antiphospholipid syndrome). Therefore, the need for effective warfarin reversal strategies remains clinically relevant. This study assessed whether the study sites’ newly implemented fixed-dose FEIBA regimen increased the proportion of patients who achieved a post-FEIBA INR ≤ 1.5 compared with the previous regimen.

## 2. Materials and Methods

This retrospective study included two hospitals within a single health care system: a 685-bed academic-affiliated medical center (>130,000 annual emergency department [ED] visits) and a 352-bed non-academic community hospital (>85,000 annual ED visits). All study measures and procedures were performed according to the Helsinki Declaration and approved by the study sites’ Institutional Review Board. This study adhered to the Strengthening the Reporting of Observational Studies in Epidemiology (STROBE) guidelines [25]. This article is a revised and expanded version of an abstract presented at a conference [26].

In 2022, the study sites updated their fixed-dose FEIBA (Takeda Pharmaceuticals, Lexington, MA, USA) protocol after an assessment of their previous dosing recommendations (INR < 5: 500 units, INR ≥ 5: 1000 units) demonstrated that 50% of patients achieved a post-FEIBA INR ≤ 1.5. Further review revealed that patients with an INR ≥ 10 were least likely to achieve post-FEIBA INR targets, prompting the development of dosing recommendations specific to this subgroup. Additionally, FEIBA doses were increased by 500 units for patients in the INR 1.6–4.9 and 5–9.9 groups, with the intent of improving the likelihood of achieving post-FEIBA INR goals while maintaining a conservative incremental adjustment to balance efficacy and safety.

Under the revised protocol, patients presenting with an INR < 5, 5–9.9, and ≥10 were recommended to receive 1000, 1500, and 2000 units, respectively (Table 1). As part of the warfarin reversal protocol, all patients were recommended to receive 10 mg of IV vitamin K, a component that remained unchanged from the prior protocol. Adherence to dosing recommendations was not mandated, and providers could order alternative doses at their clinical discretion. FEIBA is the only available PCC at the study sites.

Adult patients (≥18 years of age) with an initial INR > 1.5 who received FEIBA at a dose consistent with institutional recommendations for the management of a warfarin-associated hemorrhage were included. Patients were excluded if they were pregnant or incarcerated, received FEIBA outside institutional dosing recommendations, lacked a 6 h post-FEIBA INR, received FEIBA for reversal of an anticoagulant other than warfarin or for a non-drug-induced hemorrhage, underwent preoperative warfarin reversal in the absence of active bleeding, or received fresh frozen plasma (FFP).

Patients with an initial INR > 1.5 who received FEIBA between 2014 and 2025 were identified through a system-generated report. All patient charts in the report were reviewed beginning with those who received the old regimen to determine inclusion. Subsequently, charts of patients who received the new regimen were reviewed until the number enrolled matched that of the old regimen group.

The primary efficacy outcome was the percentage of patients in the old and new groups who achieved a post-FEIBA INR ≤ 1.5. The primary safety outcome was the rate of thromboembolic events in the old and new groups, which were monitored through discharge. A subgroup analysis evaluated the proportion of patients who achieved a post-FEIBA INR ≤ 1.5, stratified by their initial INR. The first INR level drawn following FEIBA administration was analyzed to restrict the evaluation to a single dose. Medication-related information was collected from the medication administration record (Epic Systems Corporation, Verona, WI, USA), and reversal indications were obtained from provider documentation.

Study investigators manually abstracted all medication-related information and laboratory data from the electronic medical record using a standardized abstraction tool. Two trained medical students, supervised by the principal investigator (an emergency medicine clinical pharmacist), performed data abstraction. All variables underwent dual independent abstraction to ensure accuracy, and any discrepancies were reviewed and resolved by the principal investigator.

The Mann–Whitney U test was used to compare non-normally distributed continuous variables. For selected outcomes, effect estimates were expressed as relative risks or median differences with corresponding 95% confidence intervals (95% CI). Dichotomous variables were analyzed using the Chi-Square or Fisher’s exact tests. An a priori *p*-value of <0.05 was considered statistically significant. Statistical analyses were performed using IBM SPSS Statistics for Windows, Version 31.0 (IBM Corp., Armonk, NY, USA). The sample size was limited to the number of patients who met eligibility criteria during the study period, and no a priori power calculation was conducted.

## 3. Results

Among the 533 charts screened, 497 were excluded, primarily due to receiving FEIBA for the reversal of a different anticoagulant or non-drug-induced hemorrhage (89%). Warfarin indication, medical history, anti-platelet use, and reversal indication did not differ significantly between the groups (Table 2). In the total population, patients most often received FEIBA for an intracranial hemorrhage followed by a gastrointestinal bleed. Six (33%) patients in the old group presented with a traumatic bleed, whereas 5 (28%) patients in the new group presented with a traumatic bleed (*p* = 0.72).

Patients in the new group received significantly higher total and weight-based FEIBA doses (Table 3). Fourteen (78%) patients in the old group received vitamin K, whereas 15 (83%) patients in the new group received vitamin K (*p* = 1). The median (IQR) pre-FEIBA INR in the old and new groups was 6.1 (3.1–12.9) and 5.3 (3.4–13.0), respectively [difference: −0.8 (95 CI%, −7.2 to 6.5)]. The median (IQR) post-FEIBA INR in the old and new groups was 1.6 (1.4–2.1) and 1.4 (1.2–1.5), respectively [difference: −0.2 (95% CI: −0.6 to 0.0)]. A post-FEIBA INR ≤ 1.5 was achieved in 14 (78%) patients in the new group and 9 (50%) patients in the old group (relative risk: 1.56; 95% CI, 0.91 to 2.7; *p* = 0.16). Among the four patients in the new group who did not achieve a post-FEIBA INR ≤ 1.5, post-FEIBA INR values were 1.6, 1.6, 1.8, and 2.4. The time to obtaining a post-FEIBA INR was similar between groups. One thromboembolic event occurred in the overall cohort, involving a patient in the old group with a history of Factor V Leiden. The median length of stay was comparable between groups, with a median difference of 1.1 days (95% CI, −6.1 to 5.6).

In the subgroup analysis evaluating achievement of post-FEIBA INR ≤ 1.5 according to initial INR values, no significant differences were observed between the groups among patients with initial INR values of 1.6–4.9 and 5–9.9 (Table 4). For patients with an initial INR ≥ 10, the proportion achieving a post-FEIBA INR ≤ 1.5 was significantly higher in the new group compared with the old group (86% vs. 17%, respectively).

## 4. Discussion

In this retrospective study, the proportion of patients achieving a post-FEIBA INR ≤ 1.5 did not differ significantly between groups, although median post-FEIBA INR values were lower in the new group. Subgroup analysis demonstrated that a significantly greater proportion of patients with a baseline INR ≥ 10 in the new group achieved a post-FEIBA INR ≤ 1.5. However, this finding should be considered exploratory and hypothesis-generating rather than confirmatory. Although only one thromboembolic event was observed, the study was underpowered to draw definitive conclusions regarding the safety of either intervention. Despite these limitations, this study provides additional real-world evidence regarding FEIBA dosing strategies for warfarin reversal that may inform future research and institutional practice.

**Figure 1:**
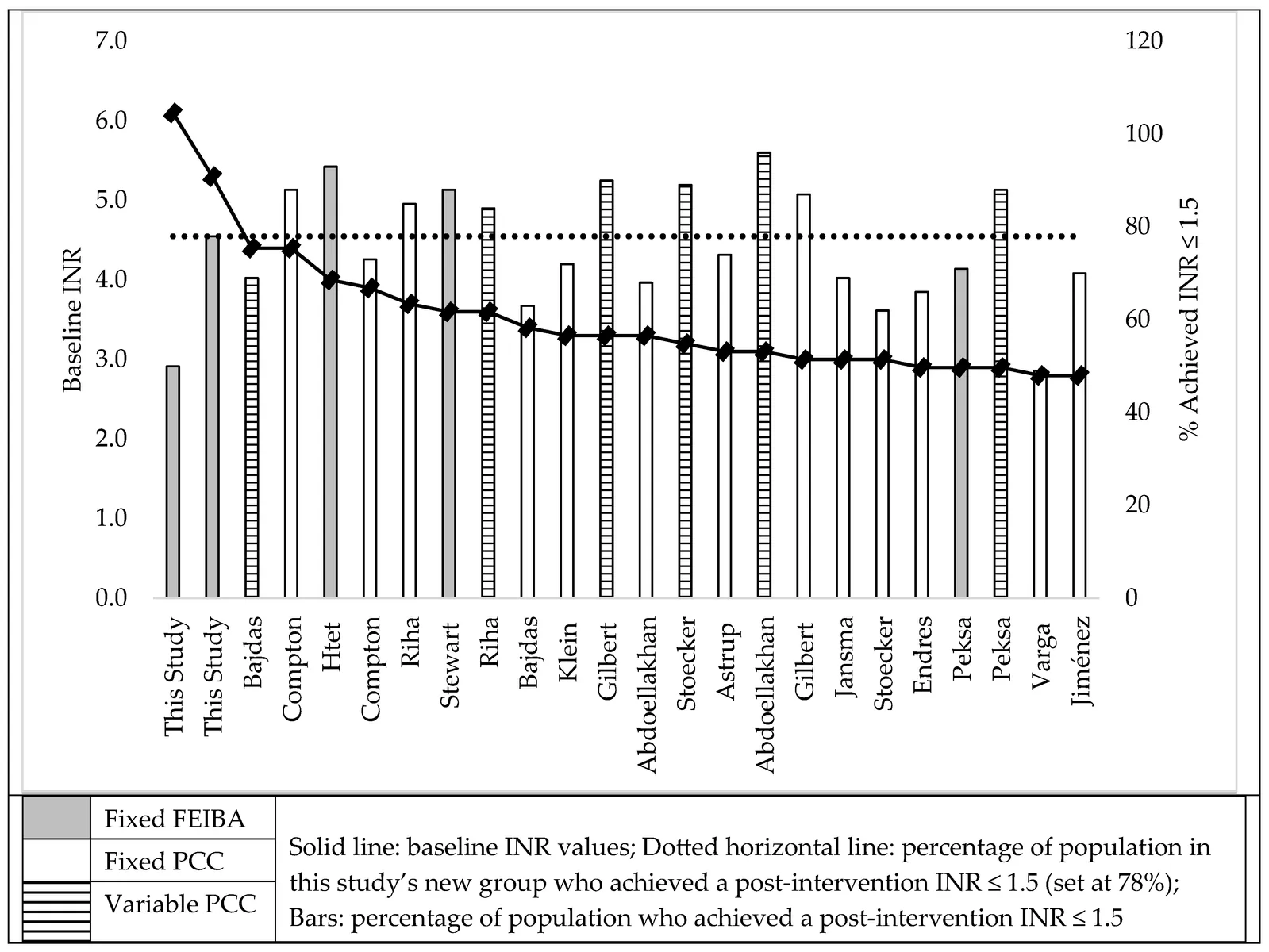
Comparison of studies targeting a post-intervention INR ≤ 1.5.

In comparison to other studies targeting a post-PCC INR ≤ 1.5, our cohort had notably higher initial INR values and did not concomitantly receive FFP (Table 5, Figure 1) [7,8,9,10,11,12,13,14,15,16,17,18,19,20,21]. Given the dose-dependent depletion of vitamin K-dependent clotting factors produced by warfarin, lower PCC doses may not provide adequate factor replacement to normalize the INR in patients with higher initial INR values. This was evident in our historical cohort, where only 50% of patients achieved the target post-FEIBA INR, prompting the dosing protocol revision. Furthermore, the current subgroup analysis reinforces this finding, as patients in the new group with an initial INR ≥ 10 were significantly more likely to achieve a post-FEIBA INR ≤ 1.5 than those in the old group with the same initial INR. While FFP contains lower levels of clotting factors than PCC, the additional factor replacement it provides may make combined FFP and PCC therapy comparable to higher PCC dosing. Despite extensive research on fixed-dose PCC regimens, our findings remain important because they underscore how initial INR levels and the concomitant use of FFP influence study outcomes; considerations that have not been highlighted in the prior literature.

To our knowledge, this is the first study evaluating our new FEIBA protocol, as published studies to date have only evaluated our former dosing protocol [9,11,19]. Two of these studies reported higher rates of patients achieving a post-FEIBA INR ≤ 1.5, but both had initial median INR values ≤ 4, and in one of these studies 37.5% of the population also received FFP [9,11]. Conversely, in another study evaluating patients with a median initial INR of 2.9, the proportion of patients who achieved a post-FEIBA INR ≤ 1.5 was less than ours and 33.7% of the population received FFP [19]. Variability in outcomes reported across fixed-dose studies is not limited to fixed-dose FEIBA studies, with inconsistencies in hemostatic effectiveness reported in non-activated fixed-dose PCC studies as well [24]. In a recent systematic review and meta-analysis, Alwakeal et al. reported that fixed-dose PCC regimens were associated with lower PCC utilization, shorter administration times, improved hemostatic outcomes, and reduced rates of mortality and thromboembolic complications compared with manufacturer-recommended dosing strategies. Among patients with warfarin-associated intracranial hemorrhages, fixed-dose regimens achieved similar rates of INR correction to less than 2 and comparable hemostatic effectiveness. However, low-certainty evidence suggested that fixed-dose regimens were less likely to achieve more stringent INR targets of less than 1.5. The authors noted substantial heterogeneity across several analyses. Differences in dosing protocols, patient populations, reversal indications, and definitions of treatment success likely contributed to this variability. Collectively, these findings underscore the need for continued investigation to better define the optimal fixed-dose reversal strategy.

Our study adds to the growing body of evidence supporting the use of fixed-dose PCC regimens, including FEIBA, which is not currently guideline recommended despite studies demonstrating its comparable efficacy to non-activated PCC [2,3,4,5,6,19,22,23]. In a study conducted by Rowe et al., a greater proportion of patients who received fixed-dose FEIBA (INR < 5: 500 units; INR ≥ 5: 1000 units) achieved the post-intervention INR goal compared to those who received manufacturer-recommended non-activated PCC dosing, but this finding did not reach statistical significance [22]. Similarly, Dietrich et al. found no significant differences in the proportion of patients who achieved the post-intervention INR goal when comparing fixed-dose FEIBA (INR < 5: 500 units; INR ≥ 5: 1000 units), fixed-dose non-activated PCC (1500 units; 2000 units if INR ≥ 7.5 or ≥100 kg), and manufacturer-recommended non-activated PCC dosing [23]. In contrast, Peksa found that significantly more patients achieved post-intervention INR goals when receiving manufacturer-recommended non-activated PCC dosing compared with fixed-dose FEIBA (INR < 5: 500 units; INR ≥ 5: 1000 units) [19]. The existing data does not definitely demonstrate one agent to be superior to the other and additional studies are warranted to determine if there is a preferred agent.

The findings of this study should be interpreted in light of several limitations, most importantly the small sample size. Although the study period was extended to account for the shift from warfarin to direct oral anticoagulants, the number of eligible patients remained limited. Consequently, subgroup analyses should be considered exploratory and hypothesis-generating rather than confirmatory. Furthermore, the multiple subgroup comparisons performed in this underpowered study increase the risk of type I error, warranting cautious interpretation of any observed differences. The revised dosing protocol was informed in part by historical data that overlapped with the old-regimen cohort included in this analysis. Therefore, findings among patients with a baseline INR ≥ 10 should be interpreted cautiously, as they do not represent a fully independent validation of the revised dosing strategy. Efficacy was assessed using INR correction, which may not necessarily reflect hemostatic effectiveness or clinical outcomes. However, this endpoint was selected because it is the most commonly reported outcome in the literature, allowing for broader contextualization of our findings. Nevertheless, direct comparisons with previously published studies remain challenging due to substantial differences in study design, interventions, and patient populations, highlighting the need for additional research. Another limitation is that the old and new protocol groups were treated during different time periods over approximately 11 years. As a result, changes in clinical practice, patient selection, or other unmeasured factors over time may have influenced the results independently of the dosing protocol. Lastly, data abstractors were not blinded to the study hypothesis, which may have introduced bias into the data collection process.

## 5. Conclusions

The new FEIBA dosing regimen was associated with a numerically higher proportion of patients achieving INR reversal, particularly among patients with a baseline INR ≥ 10, although the limited sample size warrants confirmation in larger studies. Our findings underscore how initial INR levels and the concomitant use of FFP influence study outcomes; considerations that have not been highlighted in the prior literature. Collectively, these findings contribute to the growing body of evidence supporting the use of fixed-dose PCC regimens, including FEIBA, and underscore the need for large prospective studies to further clarify the optimal fixed-dose approach and preferred PCC product for the management of warfarin-associated hemorrhages.

## Figures and Tables

**Table 1:** Old and new fixed-dose FEIBA dosing protocols.

INR	Old	New
<5	500 units	1000 units
5–9.9	1000 units	1500 units
≥10	1000 units	2000 units

**Table 2:** Baseline characteristics.

	Old	New	*p*-Value
N	18	18	-
Male, n (%)	13 (72)	10 (56)	0.48
Age, y	70 (61–81)	77 (63–87)	0.34
Weight, kg	74.3 (65.1–104.5)	75.5 (62.6–86.6)	0.42
Warfarin indication, n (%)			
Atrial fibrillation	8 (44)	10 (56)	0.5
Factor V Leiden	1 (6)	1 (6)	1
Protein S deficiency	1 (6)	0 (0)	1
Thromboembolism	7 (39)	6 (33)	0.73
Valve replacement	5 (28)	8 (44)	0.3
Past medical history, n (%)			
Cancer	0 (0)	4 (22)	0.1
Cerebrovascular accident	7 (39)	7 (39)	1
Diabetes	6 (33)	6 (33)	1
Heart failure	8 (44)	10 (56)	0.5
Hypertension	12 (67)	11 (61)	0.73
Single anti-platelet use, n (%)	9 (50)	7 (39)	0.5
Reversal indication, n (%)			
Epistaxis	0 (0)	1 (6)	1
Gastrointestinal bleed	5 (28)	5 (28)	1
Hemoptysis	0 (0)	2 (11)	0.49
Hemothorax	1 (6)	1 (6)	1
Intracranial hemorrhage	8 (44)	6 (33)	0.49
Musculoskeletal	3 (17)	1 (6)	0.6
Pericardial effusion	1 (6)	1 (6)	1
Retroperitoneal	0 (0)	1 (6)	1
Traumatic bleed, n (%)	6 (33)	5 (28)	0.72

Values are reported as medians (interquartile ranges) unless stated otherwise; Categorical variables were compared using Chi-Square or Fisher’s exact tests, as appropriate. Fisher’s exact test was used when cell counts were <5.

**Table 3:** Results.

	Old	New	Difference, 95% CI
N	18	18	-
FEIBA dose received			
Units	970 (574–1062)	1513 (1129–1933)	543 (168, 993)
Units/kg	9.6 (7.4–14.7)	20.8 (13.7–28.6)	11.2 (3.8, 19.7)
Pre-FEIBA INR	6.1 (3.1–12.9)	5.3 (3.4–13.0)	−0.8 (−7.2, 6.5)
Post-FEIBA INR	1.6 (1.4–2.1)	1.4 (1.2–1.5)	−0.2 (−0.6, 0.0)
Time to post-FEIBA administration INR, min	134 (64–194)	79 (57–112)	−55 (−111, 13)
Length of stay, d	8.4 (5.6–12.1)	9.5 (4.0–13.9)	1.1 (−6.1, 5.6)

Values are reported as medians (interquartile ranges).

**Table 4:** Achievement of post-FEIBA INR ≤ 1.5 according to baseline INR.

	Old	New	*p*-Value
Baseline INR: 1.6–4.9, n	6	7	-
Post-FEIBA INR ≤ 1.5, n (%)	5 (83)	6 (86)	1
Baseline INR: 5–9.9, n	6	4	-
Post-FEIBA INR ≤ 1.5, n (%)	3 (50)	2 (50)	1
Baseline INR ≥ 10, n	6	7	-
Post-FEIBA INR ≤ 1.5, n (%)	1 (17)	6 (86)	0.03

Categorical variables were compared using Chi-Square or Fisher’s exact tests, as appropriate. Fisher’s exact test was used when cell counts were <5.

**Table 5:** Comparison of studies targeting a post-intervention INR ≤ 1.5.

Ref.	Agent	Dosing (Units)	Baseline INR	% Goal	FFP, %
This study	aPCC	INR < 5: 500; INR ≥ 5: 1000	6.1	50	0
This study	aPCC	INR < 5: 1000; INR 5–9.9: 1500; INR ≥ 10: 2000	5.3	78	0
7	PCC	Standard dosing	4.4	69	6.3
8	PCC	4000	4.4	88	NS
9	aPCC	INR < 5: 500; INR ≥ 5: 1000	4.0	93	0
8	PCC	2000	3.9	73	NS
10	PCC	1500	3.7	85	NS
11	aPCC	INR < 5: 500; INR ≥ 5: 1000	3.6	88	37.5
10	PCC	Standard dosing	3.6	84	NS
7	PCC	INR 1.7–1.9: 500; INR 2–6: 1000	3.4	63	10.
12	PCC	1500	3.3	72	28.2
13	PCC	Standard dosing	3.3	90	23.3
14	PCC	1000	3.3	68	NS
15	PCC	Standard dosing	3.2	89	NS
16	PCC	1500	3.1	74	24.3
14	PCC	Standard dosing	3.1	96	NS
13	PCC	1500: ICH (2000: INR > 10 or >100 kg) 1000: non-ICH (1500: INR > 10 or >100 kg)	3.0	87	23.3
17	PCC	1500	3.0	69	17.2
15	PCC	1500	3.0	62	NS
18	PCC	<40 kg: 1000; 41–60 kg: 1500; 61–80 kg: 2000; 81–100 kg: 2500; >100 kg: 3000	2.9	66	21.9
19	aPCC	INR < 5: 500; INR ≥ 5: 1000	2.9	71	33.7
19	PCC	Standard dosing	2.9	88	38.4
20	PCC	1000	2.8	49	9.7
21	PCC	1000	2.8	70	NS

aPCC: activated prothrombin complex concentrates; PCC: non-activated prothrombin complex concentrates; ICH: intracranial hemorrhage; NS: not specified; FFP: fresh frozen plasma; Standard dosing: INR 2–3.9 (25 units/kg), INR 4–6 (35 units/kg), INR > 6 (50 units/kg).

## Data Availability

The original contributions presented in this study are included in the article. Further inquiries can be directed to the corresponding author.
